# Diet and Lifestyle Modifications for Fibromyalgia

**DOI:** 10.2174/0115733971274700231226075717

**Published:** 2024-04-16

**Authors:** Caroline Metyas, Tun Tun Aung, Jennifer Cheung, Marina Joseph, Andrew M. Ballester, Samy Metyas

**Affiliations:** 1 Department of Rheumatology, Covina Arthritis Clinic, 500 West San Bernadino Rd, Suite A, United States

**Keywords:** Fibromyalgia, anti-inflammatory diet, lifestyle modification, anti-oxidant diet, gluten-free diet, vitamin D

## Abstract

Fibromyalgia (FM) is a complex, widespread pain disorder characterized by symptoms such as fatigue, sleep deprivation, mental fog, mood swings, and headaches. Currently, there are only three FDA-approved medications for FM patients: duloxetine, milnacipran, and pregabalin, with outcomes frequently being inadequate.

This research team aims to investigate the effects of diet and lifestyle modifications on FM, with emphasis on anti-inflammatory diet, antioxidants, and gluten-free diets, as well as supplementation with Magnesium, CQ10, and Vitamin D, microbiome, sleep, exercise, and cognitive behavioral therapy. We reviewed the pathophysiology of certain foods that can be proinflammatory with the release of cytokines leading to activation of pain, fatigue and aggravation of the majority of Fibromyalgia symptoms. A literature review was performed by identifying FM articles published between 1994 and 2022 *via* PubMed and EMBASE databases, with particular emphasis on randomized controlled trials, meta-analysis, and evidence-based treatment guidelines. This review article was completed by a comprehensive narrative review process, in which our team systematically examined relevant scientific literature to provide a comprehensive overview of the significant role that diet and other lifestyle modifications play in mediating symptoms of Fibromyalgia.

We propose that diet modifications and lifestyle changes, such as sleep, exercise, and weight loss, can be important steps in managing FM.

## 1 INTRODUCTION

Fibromyalgia (FM) is a complex, widespread pain disorder characterized by symptoms such as fatigue, sleep deprivation, mental fog, mood swings, and headaches [1, 2]. Approximately 2% to 8% of the general population has FM, with more women being affected than men [3, 4]. Currently, there are only three FDA-approved drugs for FM patients: duloxetine, milnacipran, and pregabalin, with outcomes frequently being inadequate [5]. There are also several nonpharmacological treatments for FM, like cognitive behavior therapy (CBT) and group psychotherapy, which help with patients’ functionality [6, 7]. Acupuncture has also been shown to be effective in relieving pain for patients with FM [8]. This research team investigates diet as a potential cause of FM, which may be treated with lifestyle modifications such as sleep, exercise, and weight loss [9-11]. We performed a thorough literature search by identifying FM articles published on PubMed and Embase databases between 1994 and 2022, with emphasis on randomized controlled trials, systematic reviews, meta- analyses, and evidence-based treatment guidelines. This review article was completed by a comprehensive narrative review process, in which our team systematically examined relevant scientific literature to provide a comprehensive overview of the significant role that diet and other lifestyle modifications play in mediating symptoms of Fibromyalgia. We reviewed the pathophysiology of certain foods that can be proinflammatory with the release of cytokines leading to activation of pain, fatigue and aggravation of the majority of Fibromyalgia symptoms. We propose that changing diet and life style changes can be an important step in managing FM.

## 2 ANTI-INFLAMMATORY DIET IN FIBROMYALGIA

Lifestyle choices, namely dietary habits, largely influence the occurrence of musculoskeletal pain. In patients with FM, a lack of conclusive evidence on available treatments has made the pursuit of alternative methods of treatment more critical. One such method is dietary intervention. In most omnivorous Western diets, inflammatory nutrients, including glutamate, are pervasive, which aggravates symptoms of FM [12]. On the other hand, those who consume plant-based diets are more likely to have more controlled serum glucose and lower saturated fat and cholesterol levels, which may help alleviate some of the symptoms of FM [13]. Vegetarian and vegan diets are associated with low serum C-reactive protein (CRP) levels as compared to meat-eating diets [14]. Additionally, plant-based diets are shown to be related to decreased plasma concentrations of CRP, leukocytes, and fibrinogen [15]. Further, it has been discovered that there is a correlation between plant-based diets and low levels of oxidative stress and inflammation [16].

At Alicante University in Spain, a study was conducted using patients with FM consuming either vegetarian or omnivorous diets [17]. The study concluded that following a plant-based diet improved cholesterol, peroxidase, and fibrinogen levels, body weight, pain at rest, and other symptoms of FM [17]. Additionally, it concluded that the anti-inflammatory properties of vegetarian and vegan diets improved the maladies associated with FM, allowing FM patients who follow a plant-based diet to have a better quality of life [17].

Another dietary addition that may aid in symptom relief of FM patients includes adding olive oil to an individual’s diet. Reactive oxygen species are prevalent in patients with FM, and consuming extra virgin olive oil, which contains antioxidant phenolic compounds, may protect lipids, DNA, and proteins from oxidative damage [18]. Patients with FM who consumed extra-virgin olive oil for three weeks had a strong improvement in FM symptoms [18]. Additionally, increased consumption of ancient grains, including Khorasan, resulted in significant improvement in FM symptoms [19].

Furthermore, FM patients with GI issues may also benefit from following a low-FODMAP (fermentable oligo-di-mono-saccharides and polyols) diet, which eliminates poorly absorbed short-chain carbohydrates [20]. A study of FM patients following a low-FODMAP diet for one month concluded that eliminating these carbohydrates results in the alleviation of FM symptoms [21]. Calorie restriction, which results in lower body weight, may also alleviate FM symptoms because obesity may exacerbate pain in FM [22].

Fatigue, the aggregation of many unspecified clinical symptoms, including brain fog, headaches, joint pain, and poor sleep, is often associated with the over-activation of inflammatory mechanisms [23]. Inflammation is thought to aggravate fatigue symptoms, described by pathways including the dysfunction of natural killer cells and T/B cell memory, increased activation of nuclear factor kappa beta (NF-κB), chronic innate antiviral signaling pathway activation, and increased inflammatory markers [24-28]. Since disease-related fatigue seems to be related to a pro-inflammatory condition, dietary interventions may help decrease inflammation and fatigue. Vitamin A supplementation may be helpful as it suppresses pro-inflammatory T-cells and the gene expression of inflammatory cytokines as well as their transcription factors [29]. Supplementation with Vitamin D counterbalances NF-κB activity, which has anti-inflammatory properties [30]. Food items such as polyphenols, such as soy products, green tea, and quercetin, are also known to have anti-inflammatory properties [31-33]. Consuming probiotics may be another method of intervention as they are thought to support anti-inflammatory status by increasing dendritic and regulatory T- cells and decreasing TNF-α levels [34, 35]. Ginseng may also have anti-inflammatory properties, as one study showed that continued consumption of concentrated ginseng capsules resulted in decreased TNF-α levels and reduced fatigue [36]. Increased intake of anti-inflammatory foods rich in omega-3’s, carotenoids, and vitamins A and C, resulted in decreased inflammation and fatigue [37]. Additionally, those who followed the Mediterranean diet, eating mainly whole grains, nuts, lean meats, olive oil, fruits and vegetables, and lean fish, experienced fatigue reduction. The Mediterranean diet is rich in omega-9-mono-unsaturated fatty acid oleic acid, which is converted to eicosatrienoic acid in the body [38]. Eicosatrienoic acid is a leukotriene B4 synthesis inhibitor and may have significant anti-inflammatory properties [39].

Fatigue is one of the main symptoms often associated with the pro-inflammatory status of FM patients, which is why the implementation of anti-inflammatory diets should be emphasized for patients suffering from FM. A multi-modal lifestyle may be a successful approach in managing fatigue symptoms of FM as, even though anti-inflammatory diets may alleviate fatigue, it does not always decrease inflammatory load [40]. Therefore, it may be concluded that inflammation is not the sole aggressor of fatigue in FM patients but rather is part of a larger multi-factorial syndrome [40]. By adopting a multi-modal approach, as well as by incorporating the implementation of an anti-inflammatory diet with exercise and both pharmacological and non-pharmacological strategies, fatigue management in patients with FM may be more successful [40].

## 3 ANTIOXIDANTS IN FIBROMYALGIA

Oxidative stress has been identified as a key component of the pathophysiology of a range of conditions. The buildup of reactive oxidative species (ROS) has been shown to cause damage to cellular components and tissues through accumulating inflammatory processes and damaging physiological processes and organ systems [41]. While many studies have elucidated the role of oxidative stress in various disease states, the role ROS may play in the pathophysiology of FM has become increasingly more understood.

Oxidative stress, specifically elevated nitric oxide (NO) levels, has been linked to FM as far back as 2005 [42]. More recent studies have attempted to further examine this association *in vivo*. Plasma NO and lipid peroxidation (LPO) levels have been found to be significantly elevated in FM patients and demonstrated a positive correlation with FM severity. At the same time, antioxidant levels (catalase, superoxide dismutase, *etc.*) were found to be significantly lower in FM patients and correlated negatively with FM severity [43]. Accordingly, many studies have demonstrated the positive analgesic effects of antioxidant supplementation in FM. The Mediterranean diet has been shown to be beneficial in cardiovascular diseases (CVD) and autoimmune diseases such as multiple sclerosis through antioxidant tuning [44], largely due to the high level of antioxidants such foods contain [45]. Studies examining supplementation with various antioxidant compounds have found mixed results [46]. In particular, CoQ10 and other vitamins associated with antioxidant pathways have been shown to reduce FM-associated symptoms [47]. On the other hand, a clinical study examining the treatment of FM with alpha-lipoic acid (ALA) found no statistically significant treatment outcomes in the patients examined [48].

Another potential treatment of interest for FM is the use of ozone. Ozone therapy, which has been shown to be effective in treating various neurological, cardiovascular, and GI disorders, exerts its effects by inducing mild oxidative stress to reinvigorate innate antioxidant systems [49]. One study found significant positive effects in treating symptoms in FM patients with no reported side effects [50].

## 4 MICROBIOME AND FIBROMYALGIA

The gut microbiome is comprised of various microorganisms such as bacteria, fungi, protozoans, viruses, archaea, and helminths, which form a diverse ecosystem [51]. The composition of gut microbiota differs across individuals but remains relatively stable in an individual with only transient changes influenced by diet. Studies have shown that changes in diet, especially fiber from fruits, whole grains, vegetables, and other plant products, can significantly affect the gut microbiota. Some fiber types such as inulin, fructans and galacto-oligosaccharides are even considered to be prebiotic, defined as “a substrate selectively used by host microorganisms conferring a health benefit.” Studies have also suggested that polyphenols, found in fruits, vegetables, and cereals can confer health benefits with reduced risk of chronic diseases. On the contrary, owing to a low fiber and limited dietary diversity, a Westernized diet is associated with obesity and metabolic diseases, likely due to increased endotoxin-producing bacteria. However, it is worth noting that the induction of permanent changes in gut microbiota may require prolonged dietary changes, not just acute dietary changes, although research is currently ongoing [52].

The gut-brain axis is a hypothesis that suggests a bi-directional interplay between the microbiome and the brain, mediated in 3 ways: 1. Neurologic - the vagus nerve and the gut; 2. Endocrine - the hypothalamic-pituitary-adrenal axis and subsequent hormonal release; and 3. Immune - the release of cytokines. In patients with FM, changes in the microbiome include small intestinal bacterial overgrowth, increased gut permeability, an abundance of short-chain fatty acid-producing bacteria, and elevated bile acid metabolism (microbiome-mediated direct activation of endogenic bile acid receptors which increase nociceptor sensitization) [53]. Pain, fatigue, and cognitive symptoms that FM is known to cause are associated with microbiome alterations than other variables such as diet. Certain butyrate-producing bacteria are depleted in FM patients such as *Faecalibacterium prausnitzii*, known for its anti-nociceptive/inflammatory effects in the gut as well as enforcing the intestinal barrier. In contrast, levels of *Bacteroides uniformis* and *Prevotella copri* were elevated which are commonly seen in inflammatory arthritis [54]. In comparison, *Cropococcus comes*, another anti-inflammatory bacterial species in the gut, was found to be depleted in patients with chronic widespread pain. The researchers suggest that a high-fat diet mediates the depletion of *C. comes*, resulting in low-grade inflammation and chronic widespread pain [54]. Regarding probiotics as a treatment for FM, probiotics did not significantly improve depressive or anxiety symptoms but were found to reduce impulsive decision-making [54]. Probiotics have an indirect role in modulating levels of serotonin and dopamine *via* the gut-brain axis. A follow-up study found that taking multi-probiotics improved attention with reduced errors but did not influence memory [55]. The authors propose that probiotics may play a role in regulating neuroinflammation, thereby improving cognitive function in FM patients [56].

Despite increasing research exploring FM and the microbiome, determining whether FM causes changes in the microbiome or *vice versa* will aid in targeted treatment. However, determining a favorable microbiome composition will remain a challenge due to individualized variability and ecosystem complexity. For example, certain bacterial strains depleted in patients with FM are elevated in those with other rheumatologic diseases. In the meantime, increased fiber intake and reduction of fats and sugars remain a mainstay of FM treatment that may have health-related benefits [52].

## 5 MINERALS AND SUPPLEMENTS IN FIBROMYALGIA

Several minerals are thought to play a significant role in the management of FM symptoms. Recent studies have shown that magnesium citrate and amitriptyline supplementation can alleviate the majority of symptoms associated with FM [57]. It is theorized that magnesium-mediated inhibition of various nerve receptors leads to the alleviation of neuropathic pain [58]. In addition, many patients with FM experience symptoms of magnesium deficiency, such as reduced inspiratory muscle strength, with recent findings demonstrating declined intracellular magnesium content in such patients [59]. One contradictory review concluded that the use of magnesium supplementation in FM makes little to no difference in pain or depressive symptoms [60]. Iron supplementation has also shown promising benefits, with one study finding that treatment with iron can alleviate many hematological symptoms of FM [61]. In general, patient education and financial capabilities remain major barriers to accessing these types of dietary supplements. As advertising and supplement options continue to grow, interventions targeting such disparities are critical [61].

Coenzyme Q10 is another supplement with significant therapeutic potential in FM, specifically in relation to its role as an antioxidant. Reduced levels of CoQ10 have been identified in FM patients, and those with the deficiency who were treated with CoQ10 supplementation showed a significant reduction in symptoms [47]. A double-blinded randomized clinical trial examined the effects of CoQ10 supplementation on pain, anxiety, brain activity, oxidative stress, and inflammation in pregabalin-treated FM patients. The study found that supplementation was successful in reducing these symptoms and increasing antioxidant levels [62].

Other supplements have been identified as potential alleviators of FM symptoms due to their anti-inflammatory and antioxidant-inducing properties. These include capsaicin, ginger, and turmeric, among other bioactive compounds. However, human studies examining supplementation with these compounds are limited [63]. Due to muscle weakness associated with FM, some studies have also examined the therapeutic potential of creatine supplementation. One double-blinded randomized clinical study found that while FM patients supplementing with creatine did have improved muscle strength, no significant changes were observed in other FM symptoms [64].

## 6 GLUTEN AND FIBROMYALGIA

Up to 13% of the general population experiences non-celiac gluten sensitivity (NCGS). Both FM and NCGS are associated with inflammation and thus may have similar pathophysiology [65, 66]. There are many overlaps between FM and gluten sensitivity symptoms, such as irritable bowel, musculoskeletal pain, and lack of energy [1].

Gluten ingestion increases the expression of pro-inflammatory cytokines, such as interferon γ, interleukin 17 (IL-17), and interleukin 2 (IL-2), while decreasing the expression of anti-inflammatory cytokines in T-cells (like transforming growth factor-β and IL-10) [67]. These mediators of inflammation are also known mediators of pain. Hence, gluten may be directly associated with the pain felt by FM patients, and a gluten-free diet may limit pain. In one study, 20 patients with FM but who exhibited NCGS achieved remission of FM pain criteria, went back to work, returned to a normal life, and/or stopped using opioids when placed on a gluten-free diet (GFD) [68]. Several other studies have confirmed that a GFD improves FM symptoms [69-72]. Following a strict GFD may also decrease the number of tender points experienced and the number of prescriptions used by an FM patient [70]. These studies suggest that gluten may play a role in FM.

## 7 VITAMIN D AND FIBROMYALGIA

Vitamin D deficiency is a prevalent condition reaching epidemic levels and is currently estimated to affect more than 1 billion people worldwide [72]. In addition to its association with widespread, non-specific musculoskeletal (MSK) pain [72], hypovitaminosis D also leads to proximal muscle weakness, insufficient skeletal mineralization, and increased fall risk in the elderly [73]. Vitamin D deficiency may be suspected in cases of chronic (> 3 months) or recurrent musculoskeletal pain or, persistent fatigue or muscle weakness, particularly in those with minimal sunlight exposure [74]. While a definitive causative mechanism has not been established, current evidence suggests that vitamin D is an important regulator of pain pathways in the pathogenesis of FM. The human central nervous system (CNS), especially, has been an area of interest given the discovery of vitamin D receptors (VDR) and 1-α-hydroxylase enzymes (converts 25-hydroxyvitamin D to active 1,25-dihydroxyvitamin D) in many areas of the human brain. Vitamin D is also believed to regulate different neurotransmitters in the brain, particularly dopamine, acetylcholine, and serotonin, which are associated with dysregulation of pain processing in FM patients. Furthermore, vitamin D influences multiple inflammatory pathways implicated in chronic pain by upregulating the transforming growth factor beta 1 (TGF-β1), which suppresses other cytokines involved in the inflammatory pathway [75]. It also inhibits the synthesis of nitric oxide, known to be an important player in central pain sensitization pathways [76]. Additionally, chronic sympathetic hypersensitivity, a feature of FM, is hypothesized to be associated with low vitamin D levels in FM patients by blocking the parathyroid axis, which is responsible for regulating the body’s vitamin D levels [74].

Although several studies have suggested an association between vitamin D deficiency and FM, there is no consensus regarding the link between the two. Multiple studies have found that vitamin D deficiency contributes to FM symptoms, with some even reporting a significant correlation between lower Vitamin D levels and pain intensity [73]. Others suggest vitamin D deficiency may also be associated with mood disorders, including anxiety and depression [72]. Still, other studies have found no such correlation between vitamin D levels and FM symptoms [74, 77]. Similarly, studies involving actual vitamin D supplementation are lacking in consensus, limited in numbers, and suggest potential benefits of supplementation [75]. Vitamin D supplementation to levels greater than 30 ng/ml has been recommended as a safe and cost-effective treatment with significant therapeutic benefits in managing FM, particularly in reducing pain [75, 77]. However, these findings are again lacking in reproducibility as other studies found no improvement in FM symptoms despite vitamin D supplementation [74].

Conversely, some studies have even suggested FM may be the cause of vitamin D deficiency, as patients with FM have limited outdoor sun exposure, given their limited mobility and functional capacity [76-78]. Regardless, treatment of vitamin D deficiency is beneficial for FM patients, in whom long-term bone health and muscle strength are of great importance [75]. Vitamin D supplementation should be recommended in FM patients who are at risk for a deficiency, such as those with inadequate sun exposure, dietary intake, and obesity [75].

## 8 LIFESTYLE MODIFICATIONS, EXERCISE, WEIGHT LOSS IN FIBROMYALGIA

Exercise may be another therapy in the multimodal treatment of FM symptoms. Trials have shown that exercise interventions may reduce fatigue in FM patients. FM is characterized by anomalies in the central nervous system, such as abnormal levels of metabolites in the hippocampus of FM patients, in addition to structural and functional abnormalities [79-81]. Exercise can regulate metabolite levels and promote neurogenesis, angiogenesis, and hippocampus connectivity [82, 83]. As previously mentioned, the hypothalamic-pituitary-adrenal axis may also be altered due to sympathetic hyperactivity in the autonomic nervous system [84]. Since the sympathetic nervous system is hyperactive in FM, stress levels are high [85, 86]. Therefore, exercise may create a physiological response by decreasing this sympathetic activity and shifting towards parasympathetic activity, relaxing the muscular and nervous systems and reducing stress levels in FM patients [87, 88].

Studies have shown that low-intensity physical exercise, such as a combination of endurance training and coordination, improved pain in FM patients. Low-impact exercise is adjusted to the patient’s perception of their fatigue and pain, making it feasible to their levels of ability. EULAR, or the European Alliance of Associations for Rheumatology, strongly recommends aerobic exercise specifically [89]. It is generally low-intensity and well-tolerated by patients suffering from FM symptoms [90]. In addition to aerobic exercise, strengthening, stretching, and core-muscle exercises also had positive results for FM patients, as well as meditative exercises such as tai-chi [91].

Cognitive Behavior Therapy also referred to as CBT, is used in the treatment of Fibromyalgia as a short-term form of psychotherapy. It diverges from traditional talk therapy routes as it focuses on a dynamic change in thoughts and behaviors, rather than a revelation of past experiences and feelings [6]. Additionally, CBT has been shown to yield faster improvements than traditional psychotherapy, demonstrating its efficacy between 10-20 sessions [6]. Since the development of CBT as a therapy form, it has expanded to be useful in treating those who suffer from chronic pain, including those with FM. Research has indicated that, in most cases, CBT as a treatment for FM resulted in lasting improvements in patients’ behaviors relating to pain, self-confidence, as well as wellness and physical practices [6]. Results were most prominent in managing the symptoms of those with juvenile FM [6].

Acupuncture is another nonpharmacological intervention that may alleviate FM symptoms in patients experiencing chronic widespread pain and improve quality of life. Due to its analgesic effects, acupuncture may activate the peripheral and central pain control systems by releasing endogenous opioid and non-opioid compounds [8]. These include norepinephrine, serotonin, dynorphins, beta-endorphins, enkephalins, and gamma-aminobutyric acid [8]. In completing randomized controlled trials on patients with FM and analyzing them against a control group who received sham acupuncture, researchers have concluded that acupuncture is significant in reducing pain in patients with FM [8]. This was completed *via* a meta-analysis performed by the Cochrane systematic review method and demonstrates that acupuncture is a safe and effective method in reducing pain and, therefore, increasing quality of life in those with FM [8].

Sleep is another effective modality in managing FM symptoms. In addition to research indicating that the dominant pathophysiology in patients with FM is dysfunctional processing of pain, neuroimaging research has demonstrated a reduced tolerance for pressure-induced pain [9]. This phenomenon is mediated by sleep quality. It has been found that patients experiencing this phenomenon have abnormal alpha rhythms and decreased short-wave sleep [9]. This indicates that they may be awake during the non-REM cycles of their sleep [9]. As a result of poor sleep, FM symptoms may be exacerbated, including fatigue, tenderness, and myalgia [9]. These results have led researchers to postulate that poor sleep may be pathogenic rather than simply the aftermath of experiencing pain [9]. Additionally, research has demonstrated that inadequate sleep is a risk factor for chronic pain symptoms as it inhibits pathways of descending pain-inhibition, which plays a major role in pain management [9]. Therefore, clinical trials have concluded that in order to reduce pain and fatigue symptoms in FM patients, improving sleep quality is important as it is an important pathogenic trigger in FM [9].

Weight loss is also recommended as an important treatment modality for FM patients [92]. Obesity is widely regarded as a pro-inflammatory state, and leptin, an appetite-regulatory hormone produced by white adipose tissue, is being studied as a link between obesity and inflammation [93, 94]. Increased body mass index (BMI) is not only associated with higher levels of leptin but increased pain sensitivity. Additionally, leptin levels may be increased in FM patients [93]. Proposed mechanisms of leptin-induced inflammation include interleukin-6 (IL-6) production as well as the provocation of macrophages, which lead to increased pro-nociceptive factors that process pain [94]. Higher BMI may also contribute to increased pain sensitivity through higher mechanical loads from having to bear extra weight. Studies have shown that weight loss in obese patients with FM leads to significant improvements in depression, sleep quality, tender-point count, and overall quality of life. Furthermore, weight loss is also shown to reduce pro-inflammatory cytokines such as IL-6 and CRP, leading to a reduction in pain sensitivity. Lifestyle modifications to lower leptin levels, such as physical activity, weight loss, and caloric restriction, should be considered in managing FM patients [94].

Recently, Low-Level Laser Therapy (LLLT) has emerged as an FDA-approved, non-invasive treatment modality in fibromyalgia patients. LLLT has been shown to improve pain severity, anxiety, depression, fatigue, stiffness and number of tender points in FM patients, particularly those who are not able to engage in regular exercise. LLLT is believed to help reduce cell inflammation and death by its action on mitochondrial cytochrome c oxidase, thereby increasing the production of adenosine triphosphate (ATP) and reducing the reactive oxygen species levels. Other hypotheses include anti-inflammatory effects through the reduction of prostaglandin-2 and cyclooxygenase-2 levels, vasodilatory effects through an increase in nitric oxide production and proliferation of connective tissue cells. LLLT should be considered as a safe, effective treatment in the management of FM patients [95].

## CONCLUSION

Fibromyalgia is a complex, widespread pain disorder often associated with other chronic conditions. A multidisciplinary approach, including both pharmacological and non-pharmacological modalities, is often required in managing FM patients. Current available pharmacological options appear to be limited in efficacy and directed more towards palliation of symptoms and alternative therapies such as CBT, sleep hygiene, acupuncture, and thermal and light therapies may be helpful adjuncts. Behavioral modifications, including diet, exercise and weight loss, should be implemented as part of the multidimensional approach. Dietary fiber from fruits, whole grains, vegetables, and other plant products may confer health benefits and reduce the burden of FM by altering the gut microbiota. Additionally, supplementation with probiotics may improve cognitive function in FM patients. Available evidence also suggests potential benefits of introducing an anti-inflammatory and gluten-free diet, addressing Vitamin D deficiencies, as well as supplementation with Magnesium and Coenzyme Q10. These non-pharmacological modalities play an important role in the successful management of FM and should be considered as part of a patient-centered, multidisciplinary approach.

## AUTHORS’ CONTRIBUTIONS

All authors have contributed to designing the study, collecting and analyzing, interpreting data, and preparing and revising the manuscript. All co-authors have approved the final version of the manuscript.

## LIST OF ABBREVIATIONS

ATP: Adenosine Triphosphate
BMI: Body Mass Index
CRP: C-Reactive Protein
FM: Fibromyalgia
LLLT: Low-Level Laser Therapy
